# Brassinosteroids-Induced Systemic Stress Tolerance was Associated with Increased Transcripts of Several Defence-Related Genes in the Phloem in *Cucumis sativus*

**DOI:** 10.1371/journal.pone.0066582

**Published:** 2013-06-19

**Authors:** Pingfang Li, Li Chen, Yanhong Zhou, Xiaojian Xia, Kai Shi, Zhixiang Chen, Jingquan Yu

**Affiliations:** 1 Department of Horticulture, Zhejiang University, Hangzhou, People’s Republic of China; 2 Key Laboratory of Horticultural Plants Growth, Development and Quality Improvement, Ministry of Agricultural, Hangzhou, People’s Republic of China; 3 Department of Botany and Plant Pathology, Purdue University, West Lafayette, Indiana, United States of America; Instituto de Biología Molecular y Celular de Plantas, Spain

## Abstract

Brassinosteroids (BRs), a group of naturally occurring plant steroidal compounds, are essential for plant growth, development and stress tolerance. Recent studies showed that BRs could induce systemic tolerance to biotic and abiotic stresses; however, the molecular mechanisms by which BRs signals lead to responses in the whole plant are largely unknown. In this study, 24-epibrassinosteroid (EBR)-induced systemic tolerance in *Cucumis sativus* L. cv. Jinyan No. 4 was analyzed through the assessment of symptoms of photooxidative stress by chlorophyll fluorescence imaging pulse amplitude modulation. Expression of defense/stress related genes were induced in both treated local leaves and untreated systemic leaves by local EBR application. With the suppressive subtractive hybridization (SSH) library using cDNA from the phloem sap of EBR-treated plants as the tester and distilled water (DW)-treated plants as the driver, 14 transcripts out of 260 clones were identified. Quantitative Real Time-Polymerase Chain Reaction (RT-qPCR) validated the specific up-regulation of these transcripts. Of the differentially expressed transcripts with known functions, transcripts for the selected four cDNAs, which encode an auxin-responsive protein (IAA14), a putative ankyrin-repeat protein, an F-box protein (PP2), and a major latex, pathogenesis-related (MLP)-like protein, were induced in local leaves, systemic leaves and roots after foliar application of EBR onto mature leaves. Our results demonstrated that EBR-induced systemic tolerance is accompanied with increased transcript of genes in the defense response in other organs. The potential role of phloem mRNAs as signaling components in mediating BR-regulated systemic resistance is discussed.

## Introduction

Brassinosteroids (BRs), a group of naturally occurring plant steroidal compounds, are essential for plant growth and development [1]. They have also been implicated to confer tolerance against a wide spectrum of biotic and abiotic stresses, such as oxidative, thermal, salinity, heavy metal stresses and those caused by pesticides and pathogen attack in many plant species [2]–[5]. Although a large body of evidence has shown that BRs do not undergo long-distance transport between different organs [6], several studies, including ours, showed that they could induce systemic tolerance against both biotic and abiotic stresses [7], [8]. It has been reported that BRs may have an indirect role in long-distance signaling through their effects on other hormones, such as auxins or polyamines [9], [10]. In tobacco, BRs function in an innate immunity system that is distinct from systemic acquired resistance (SAR) and wound-inducible disease resistance [11]. Foliar application of brassinolide reduces the nodule number in a super-nodulating soybean mutant [12]. Further study has indicated that BRs regulate root nodule formation through a change of the polyamine content [10]. Recently, Ding has found that the foliar application of 24-epibrassinosteroid (EBR) enhances the root resistance to *Fusarium oxysporum* in cucumber plants [8]. Furthermore, Xia and others provided evidence that local EBR treatment activated continuous production of H_2_O_2_ by triggering NADPH and thus mediated systemic stress tolerance in cucumber [7]. However the long-distance molecules and related signal transduction cascades involved in the BR-mediated systemic effect remain largely unknown.

Phloem, consisting of companion cell and sieve element, which is also called the sieve-tube system, is the conduit for the transport of photoassimilates, including carbohydrates, amino acids and other nutrients, from source to sink organs [13]. Recently, numerous mRNAs, small RNAs and proteins have been found to be present in the phloem translocation stream and function as components of a long-distance signaling network [14], [15]. These proteins and RNAs have been shown to play important roles in the coordination of different organs in floral induction, tuberization, resource allocation, nutrient signaling and pathogenic defense responses [16]. For example, in tomato, wild-type scion-leaf development was shown to change due to the long distance transport of a homeobox fusion transcript *PYROPHOSPHATE-DEPENDENT PHOSPHOFRUCTOKINASE (PFP)-LeT6*, after grafting onto the dominant mutant *Mouse-ear* (*Me*) [17]. RNA for *GAI* which is involved in GA signaling has also been demonstrated to regulate leaf development through their long-distance trafficking in transgenic tomato plants [18], and in potato, the movement of BEL1 transcription factor *StBEL5* to the stolon tips has been implicated in the enhancement of tuber formation [19]. It is, therefore, highly possible that phloem mRNAs may function as signaling components that mediate BR-regulated systemic resistance.

We were particularly interested in studying the up-regulated mRNAs in phloem sap caused by BRs. Cucumber (*Cucumis sativus* ), a cucurbit plant, is an ideal species for studying vascular biology because of its distinct vascular bundles and easiness in collecting phloem sap by exudation. To test the above hypothesis, we first analyzed EBR-induced systemic stress tolerance through the assessment of symptoms of photooxidative stress by chlorophyll fluorescence imaging pulse amplitude modulation. Then a suppressive subtractive hybridization (SSH) library with RNA extracted from the phloem sap of distilled water (DW)-treated cucumber as the driver, and EBR-treated as the tester was established to detect the mRNA molecules induced by EBR. mRNAs associated with transcription, protein translation, transport and defense responses were identified. Among them, four identified transcripts involved in defense responses were further studied for their expression in local and distal organs. The results are discussed with the proposal that phloem mRNAs may function as long-distance signaling components involved in the BR-induced systemic resistance in cucumber.

## Materials and Methods

### Plant Material and Experimental Design

Cucumber (*Cucumis sativus* L. cv. Jinyan No. 4) seeds were sown in a growth medium containing a mixture of soil and perlite (1∶1, v/v). Seedlings with two cotyledons were transplanted into containers (40 cm×25 cm×15 cm) filled with Hoagland’s nutrient solution. Plants were grown in a greenhouse with a photoperiod of 14 h, average day/night temperatures of 25/18°C, and a maximum photosynthetic photon flux density (PPFD) of 1000 µmol m^−2^ s^−1^. Three experiments were carried out to investigate BR-induced changes in stress tolerance and gene transcript in the phloem sap, local and systemic leaves, and roots.

#### Experiment 1

To determine the systemic effects of BR on tolerance against photooxidative stress and the transcript level of defense-related genes, the 5^th^ leaf from the bottom of a plant at the seven-leaf stage was pretreated with 0.2 µM EBR (Sigma) or DW. Twenty four hours later, the 6^th^ leaf was sprayed with 10 µM paraquat (PQ; Sigma) and stress tolerance was measured on the basis of changes in the maximal photochemical quantum efficiency of photosystem II (Fv/Fm) at 24 h after PQ treatment. Chlorophyll fluorescence was determined with imaging pulse amplitude modulation fluorometer (IMAG-MAXI; Heinz Walz). For the measurement of Fv/Fm, plants were dark-adapted for 30 min. Minimal fluorescence (Fo) was measured during the weak measuring pulses and maximal fluorescence (Fm) was measured during a 0.8-s pulse light exposure to a PPFD of ca. 4000 µmol m^−2^ s^−1^. Fv/Fm was determined with the whole leaf as the area of interest [5]. The 5^th^ and 6^th^ leaves were sampled at different time points after EBR treatment for gene expression analysis.

#### Experiment 2

To investigate EBR-induced transcript profile changes in cucumber phloem sap by SSH library and to confirm the up-regulation of the transcripts detected by SSH library, plants at the seven-leaf stage were used for phloem sap collection. Mature leaves were treated with 0.2 µM EBR (Sigma, USA) or DW at 8 am. The phloem sap was collected at various time intervals (11 am, 2 pm, 5 pm and 8 am in the next morning) from the EBR- and DW- treated plants at the same time. The stem part close to apex and mature leaf petioles were cut, and the phloem sap was collected, as previously described [20], [21]. Briefly, stems or petioles were excised with a sterile razor blade and the cut surface was then blotted with sterile filter paper (3 MM; Whatman, Maidstone, UK) for several times. Phloem sap exuded thereafter was collected using sterile micropipette tips (200 µL) and immediately mixed with 500 µL of TRIzol reagent (Invitrogen, Carlsbad, CA) for phloem sap RNA extraction. Ten plants with ca. seven leaves for each treatment at each time interval were used, and 200 µL of the phloem sap was collected at each time interval. Phloem sap from different time intervals was pooled together for RNA extraction.

#### Experiment 3

To determine the expression of genes identified from the SSH library in the local and systemic leaves and roots, the 5^th^ leaf at the seven-leaf stage were pretreated with 0.2 µM EBR (Sigma, USA) or DW. The 5^th^ and the 6^th^ leaves were sampled at 0.5, 1, 3 and 6 h later, respectively. Meanwhile, the roots were also sampled at 6, 12, and 24 h after treatment. The samples were stored at −80°C until the RNA extraction.

### RNA Isolation from Cucumber Phloem Sap and cDNA Synthesis

Each aliquot (200 µl) of phloem sap was mixed with 500 µl TRIZOL reagent (Invitrogen, Carlsbad, CA, USA). Proteins were then extracted twice with chloroform. The RNA contained in the aqueous phase was precipitated with 2.5 volumes of cold ethanol, centrifuged at 4°C for 45 minutes (min), and then resuspended in diethyl pyrocarbonate-treated water. Total RNA was purified using the RNeasy Mini Kit (Qiagen, Hilden, Germany).

Phloem-sap-derived cDNA from the DW- and EBR-treated plants was amplified by long-distance (LD)-PCR and then used directly for SSH. The SSH library was constructed according to the manual of the Super SMART PCR cDNA Synthesis Kit (Clontech, Palo Alto, CA, USA). cDNA from the control phloem sap in DW-treated plants was used as the driver, while cDNA from the phloem sap in EBR-treated plants was used as the tester. The PCR products from the second amplification were cloned into the pGEM-T easy vector (Promega, Madison, WI, USA). Recombinant plasmids harboring the subtracted cDNA from phloem sap RNA were analyzed by PCR. Independent clones (260) were randomly selected, sequenced (Sangon, Shanghai, China) and then were subjected to BLASTN searches in the Cucurbit Genomics Database (CuGenDB): http://www.icugi.org/cgi-bin/ICuGI/genome/blast.cgi.

### Total RNA Extraction and RT-qPCR for Gene Expression Analysis

Total RNA from the phloem sap, leaves and roots of cucumber was extracted using TRIZOL reagent (Invitrogen, Carlsbad, CA, USA), according to the manufacturer’s instructions. After extraction, total RNA was dissolved in diethyl pyrocarbonate-treated water. The first-strand cDNA used as the template for RT-qPCR was synthesized using a RevertAid™ First-strand cDNA Synthesis Kit (Fermentas, USA ) from 2 µg of total RNA that was purified by the RNeasy Mini Kit (Qiagen, Hilden, Germany). Gene-specific primers for the RT-qPCR were designed based on EST sequences, which are shown in Table S1. The RT-qPCR was performed with an iCycler iQ Multicolor Real-time PCR Detection System (Bio-Rad, Hercules, CA, USA). Each reaction (20 µl total volume) consisted of 10 µl iQ SYBR Green Supermix, 1 µl of diluted cDNA and 0.1 µM of forward and reserve primers. The PCR cycling conditions were as follows: 95°C for 3 min and 40 cycles of 95°C for 10 seconds (sec) and 58°C for 45 sec. Fluorescence data were collected during the 58°C step. The cucumber *actin* gene was used as an internal control. The relative gene expression was calculated, as previously described [22].

### Statistical Analysis

Plants were arranged in three randomized blocks with three replicates per treatment. The mean values of three replicates were compared using Tukey’s test (P≤0.05).

## Results

### EBR Induces Systemic Stress Tolerance in Cucumber Plants

**Figure 1:**
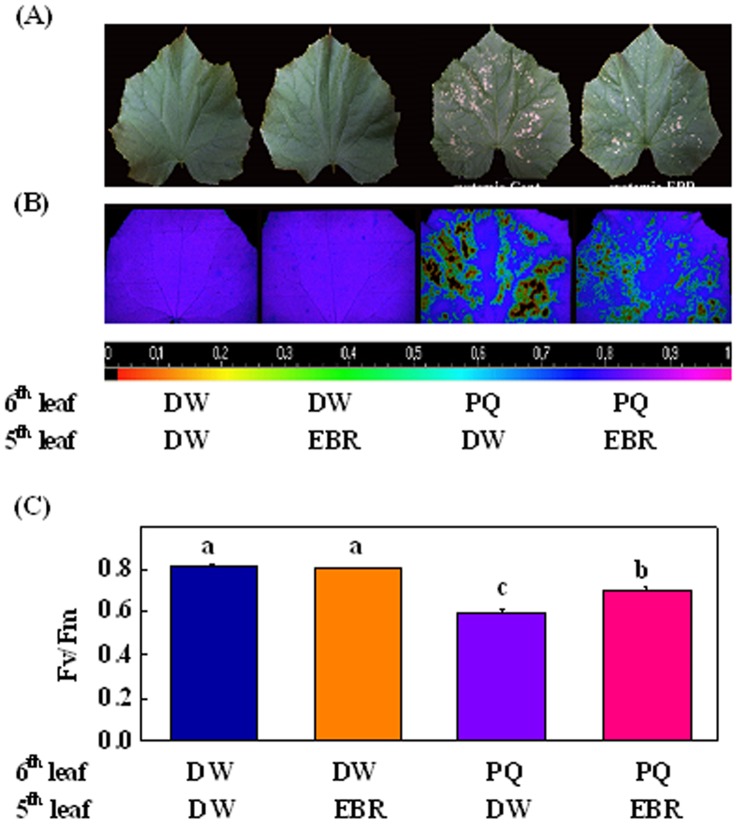
EBR induced systemic stress tolerance in cucumber plants. (A) The 5^th^ leaf of cucumber seedlings corresponding to the local treated leaf at the seven-leaf stage was pretreated with distilled water (DW) or 0.2 µM EBR; 24 h later the upper leaf (6^th^) corresponding to the systemic leaf was exposed to 10 µM paraquat (PQ), photographs were taken at 24 h after PQ treatment. (B) The maximal photochemical quantum efficiency of photosystem II (Fv/Fm) was monitored at 24 h after PQ treatment with imaging pulse amplitude modulation (PAM) to assess the changes in tolerance. (C) Fv/Fm measurement of systemic leaves. Data represent the means of five replicates ± SD. Bars sharing the same letters are not significantly different within the treatment (*P*≤0.05).

Brassinosteroids are known for their immobility in plants. To determine whether BRs could induce systemic stress tolerance, the 5^th^ leaf (defined as local leaf) of a plant was pretreated with DW or 0.2 µM EBR whilst the 6^th^ leaf (defined as systemic leaf) was sprayed with DW or 10 µM PQ and finally, Fv/Fm was determined at 24 h after the PQ treatment. Exposure of the leaf to PQ resulted in leaf bleaching but bleaching at the 6^th^ leaf was significantly alleviated by the EBR application at the 5^th^ leaf (Figure 1A). Fv/Fm did not change with the DW or EBR treatment in the absence of PQ treatment (Figure 1B, C). Exposure to PQ led to sharp decreases in Fv/Fm in all treatments. The declines in Fv/Fm at 6^th^ leaf, however, were significantly alleviated by the EBR treatment at 5^th^ leaf. Together, these results demonstrated that EBR treatment could induce systemic stress tolerance in cucumber plants.

### EBR Induces Expression of Defense/stress Related Genes in Local and Systemic Leaves

**Figure 2:**
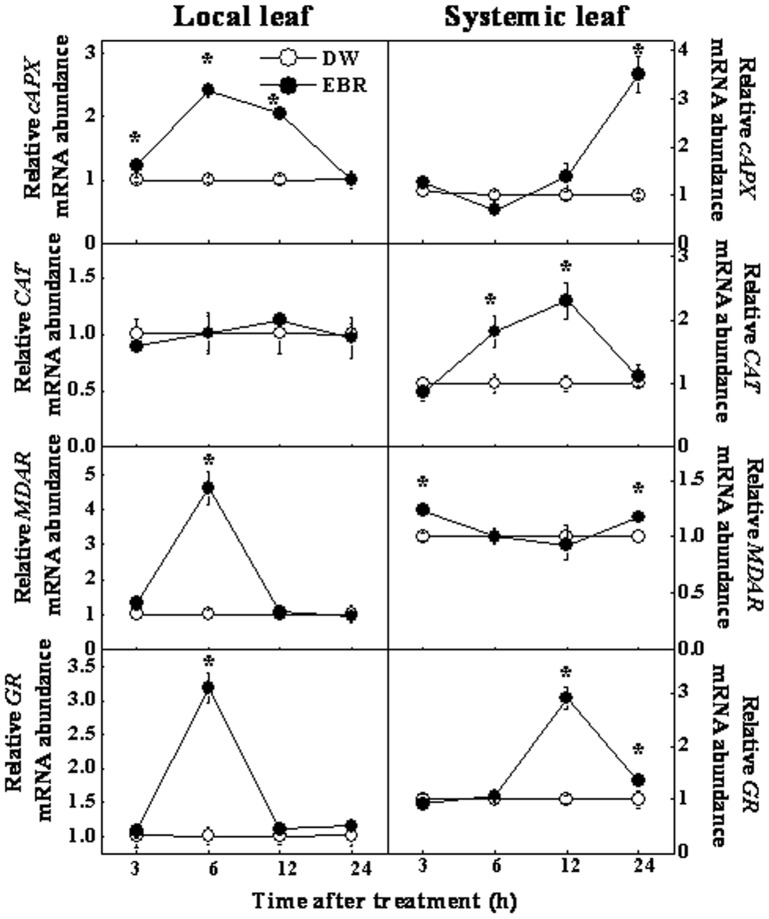
Expression analysis of defense/stress related genes in local and systemic cucumber leaves. The 5^th^ leaf of cucumber seedlings at the seven-leaf stage was pretreated with distilled water (DW) or 0.2 µM EBR, the 5^th^ leaf corresponding to the local treated leaf and the 6^th^ leaf corresponding to the systemic leaf were harvested at 3, 6, 12, 24 h after EBR treatment. Relative mRNA abundances of *cAPX*, *CAT*, *MDAR*, *GR* in EBR-treated local and untreated systemic leaves were determined by RT-qPCR analysis. Data represent the means of three replicates ± SD. Asterisks (*) indicate a significant difference from the untreated control at *P*≤0.05 according to Tukey’s test.

To further study the effects of EBR on the induction of tolerance to oxidative stress in EBR-treated local leaf (5^th^) and EBR-untreated systemic leaf (6^th^), we performed a time-course analysis of the expression of a subset of defense/stress related genes involved in biotic and abiotic stresses after EBR application on the 5^th^ leaf (Figure 2). In the local leaf, the transcript level of *cytosolic Ascobate peroxidase* (*cAPX*), *Monodehydroascorbate reductase* (*MDAR*) and *Gutathione reductase* (*GR*) increased significantly at 6 h after EBR treatment, then returned to control levels at 24 h. Interestingly, in the systemic leaf, we observed noticeable changes in the transcript abundance of some of the antioxidant related genes as well. The *cAPX*, *Catalase* (*CAT*) and *GR* expression was induced to a high level between 12 to 24 h upon EBR treatment, this induction, however, was a little later than that in local leaf. These results suggested that foliar application of EBR not only induced the expression of defense/stress related genes in the local leaf, but also in the systemic leaf in cucumber.

### The Identification of EBR-up-regulated Transcripts in Cucumber Phloem Sap by SSH Library Analysis

To determine whether EBR treatment induced changes in the phloem mRNAs, the phloem transcript profile was analyzed by constructing an SSH library. Phloem sap was collected at 3, 6, 9 and 24 h time intervals following the foliar application of DW or EBR on fully expanded, mature cucumber leaves. The DW treatment was set as the driver and the EBR treatment as the tester. A total of 686 clones were obtained, and 260 clones were randomly sequenced, in which there was a high level of redundancy in this population. Bioinformatic analysis revealed that there were only 14 unique clones (Table 1).

**Table 1:** Differential population of the mRNA transcripts induced by 24-epibrassinosteroid (EBR) in phloem sap of cucumber plants.

Clone name	Accession Number	Annotation	Best homologs in *Cucumis sativus*	*E*-value	GO biological process
Cs564	JG391944	DNA binding/methylated histone residue binding	Csa009901	3.00E-24	regulation transcription
Cs74	JG391952	Zinc finger CCCH-type with G patch domain-containing protein	Csa016350	5.00E-58	regulation transcription
Cs642	JG391945	40S ribosomal protein S7	Csa004617	1.00E-78	translation
Cs140	JG391953	60S ribosomal protein L38	Csa007567	1.00E-16	translation
Cs418	JG391947	tRNA-binding region domain-containing protein	Csa016058	1.00E-68	translation
Cs60	JG391942	ATG8C (autophagy 8c); microtubule binding	Csa026450	4.00E-61	protein transport
Cs623	JG391955	MLP-like protein 328	Csa010594	0	defense response
Cs579	JG391948	Ankyrin repeat domain-containing protein 2	Csa013665	4.00E-48	defense response
Cs453	JG391949	Auxin-responsive protein IAA14	Csa000125	3.00E-57	response to wounding
Cs594	JG391950	F-box protein PP2-A14	Csa012410	0	response to wounding
Cs681	JG391943	Yippee putative zinc-binding protein	Csa005548	1.00E-101	unknown
Cs658	JG391946	Unknown	Csa003458	1.00E-112	
Cs526	JG391954	Unknown	Csa003457	1.00E-116	
Cs302	JG391951	Unknown	Csa012053	6.00E-67	

EBR-induced changes in the population of mRNAs in the phloem sap of cucumber plants were determined by suppressive subtractive hybridization (SSH) methods. Mature leaves of seven-leaf stage plants were treated with distilled water (DW) or 0.2 µM EBR and phloem sap was collected from stem cut close to apex and at petioles during various time intervals (3, 6, 9 and 24 h) after treatment. RNA was extracted from pooled samples for use in SSH analysis. Data presented reflects the mRNA species up-regulated in EBR-treated vs DW-treated phloem sap.

The bioinformatic analysis was performed to classify these up-regulated RNA species, according to the predicted function of the encoded protein (Table 1). Of the 14 clones obtained, two clones Cs564 and Cs74 encode a putative DNA-binding protein and a zinc finger protein, respectively, which are related with transcription regulation. Three clones Cs642, Cs140 and Cs418 encode a 40S ribosomal protein, a 60S ribosomal protein and a tRNA-binding protein, respectively, which are related with protein translation. Cs60 is predicted to encode a microtubule-binding protein, which is involved in protein transport. Most relevant to our study, four clones were predicted to encode proteins related with defense responses, which included a major latex, pathogenesis-related (MLP)-like protein (Cs623), an ankyrin-repeat protein (Cs579), an F-box protein (Cs594) and an auxin response protein, IAA14 (Cs453). Further sequence alignment of Cs453 with melon phloem sap transcripts by ClustalW2 showed that it has very high similarity (max identities are 97%) with F308 (accession number EB715302) which has been identified as a phloem mobile transcript by grafting experiments in melon plants [23]. In the other clones, Cs681 encoded a putative Yippee-like zinc-binding protein, which has an undescribed biological function. Lastly, three clones, Cs658, Cs526, and Cs302, had no significant similarity to described proteins. By this analysis, we identified four genes that are related with defense responses, which may imply that they are possibly involved in EBR-regulated systemic resistance.

### Confirmation of the Up-regulation of EBR-induced Genes in Cucumber Phloem Sap

**Figure 3:**
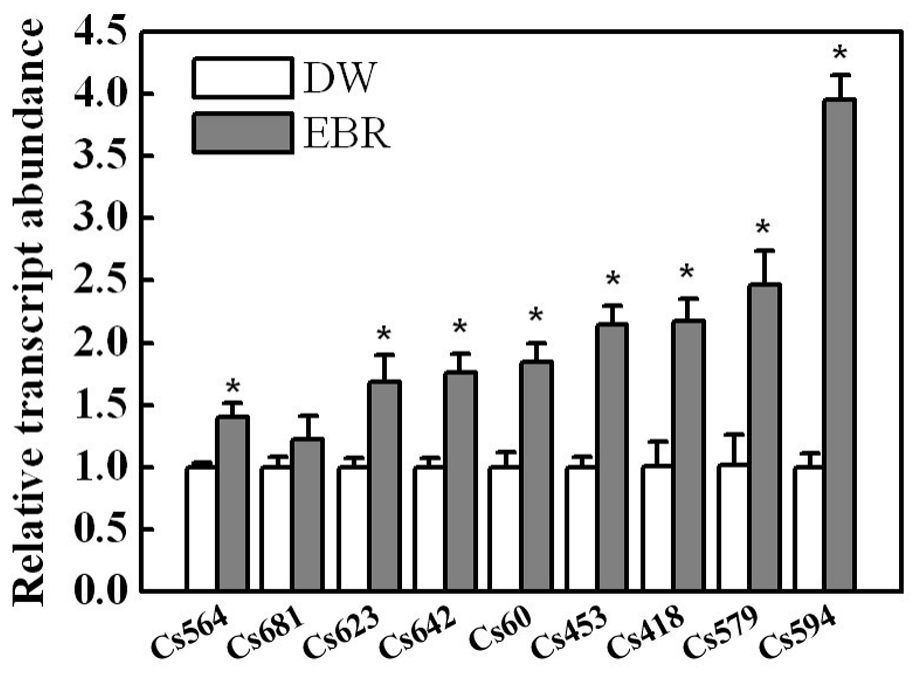
Validation of the up-regulated transcripts present in the SSH library by using RT-qPCR. Phloem sap was collected from stem cut close to apex and at petioles during various time intervals (3, 6, 9 and 24 h) after foliar EBR treatment. RNA was extracted from the pooled samples and relative mRNA abundances of transcripts obtained from the SSH library were determined by RT-qPCR. The data were obtained from three separate replicates, each value in the graph shows means ± SD. Asterisks (*) indicate a significant difference from the untreated control at *P*≤0.05 according to Tukey’s test.

RT-qPCR was then performed to verify the up-regulation of the transcripts obtained from the SSH library. Nine clones were selected for the additional RT-qPCR analysis (Figure 3). The results indicated that the transcript levels of Cs623 (coding for an MLP-like protein), Cs60 (coding for a microtubule-binding protein), Cs642 (coding for a ribosomal protein) and Cs564 (coding for a DNA-binding protein) increased by 1.5–2-fold in the EBR-treated phloem sap. Additionally, the up-regulated defense-related transcripts, Cs453 (coding for IAA14), Cs579 (coding for a putative ankyrin-repeat protein), and Cs594 (coding for an F-box family protein), were shown to be increased by 2–4-fold. Transcript Cs681 (coding for a putative Yippee-like zinc binding protein) did not increase significantly (p-value≤0.05), this may be caused by the variation between different batches of samples. Above all, these results demonstrated that the SSH library produced reliable results.

### Time-course of Gene Transcripts in Local and Systemic Leaf and Distal Organs after EBR Treatment

**Figure 4:**
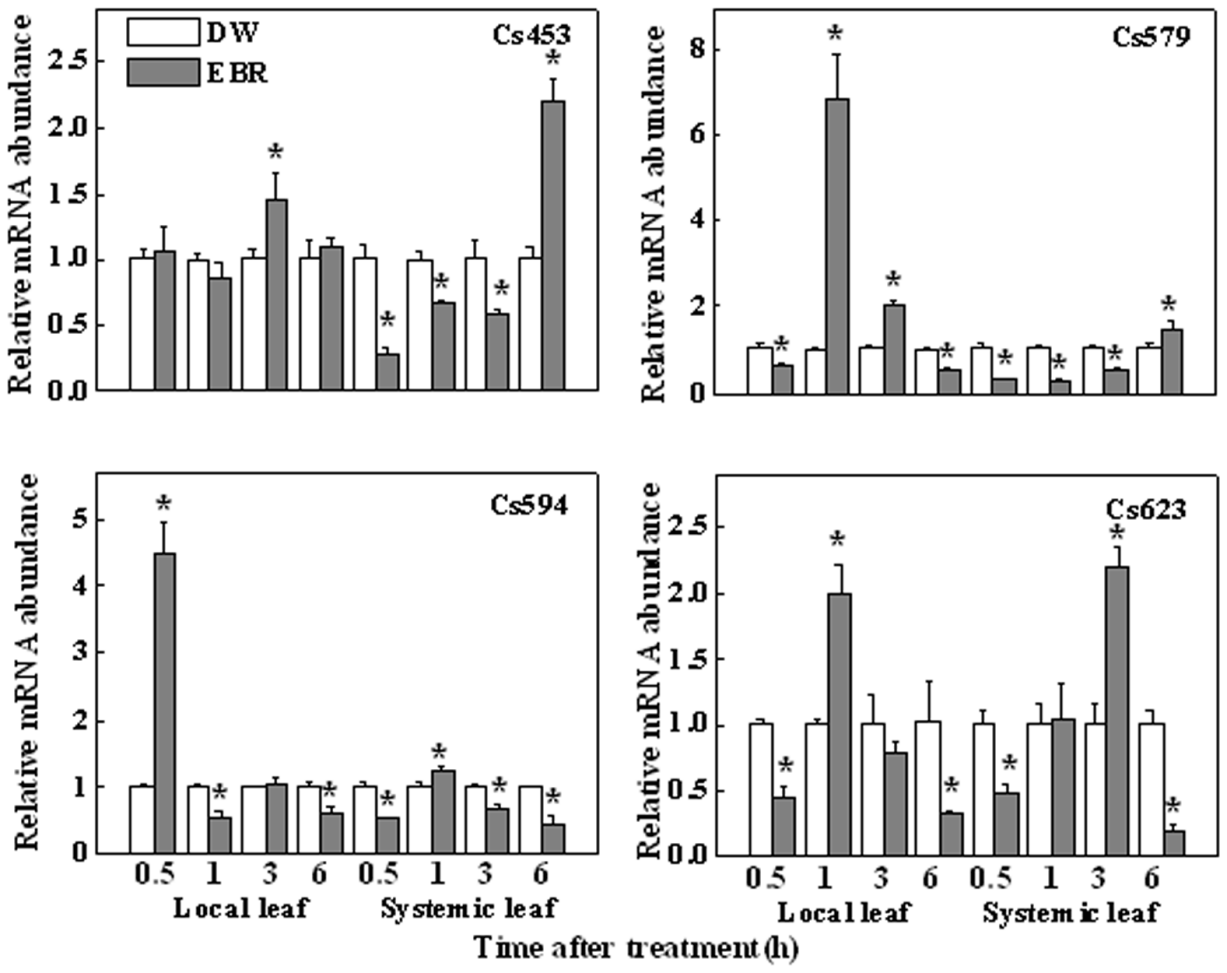
Expression analysis of defense related transcripts obtained from SSH library in cucumber leaves. The 5^th^ leaf of cucumber seedlings at the seven-leaf stage was pretreated with distilled water (DW) or 0.2 µM EBR, the 5^th^ leaf corresponding to the local treated leaf and the 6^th^ leaf corresponding to the systemic leaf were harvested at 0.5, 1, 3 and 6 h after EBR treatment. Relative mRNA abundances of Cs453, Cs579, Cs594 and Cs623 obtained from the SSH library were determined in local and systemic leaves by RT-qPCR analysis. The data were obtained from three separate replicates, and each value in the graph shows means ± SD. Asterisks (*) indicate a significant difference from the untreated control at *P*≤0.05 according to Tukey’s test.

**Figure 5:**
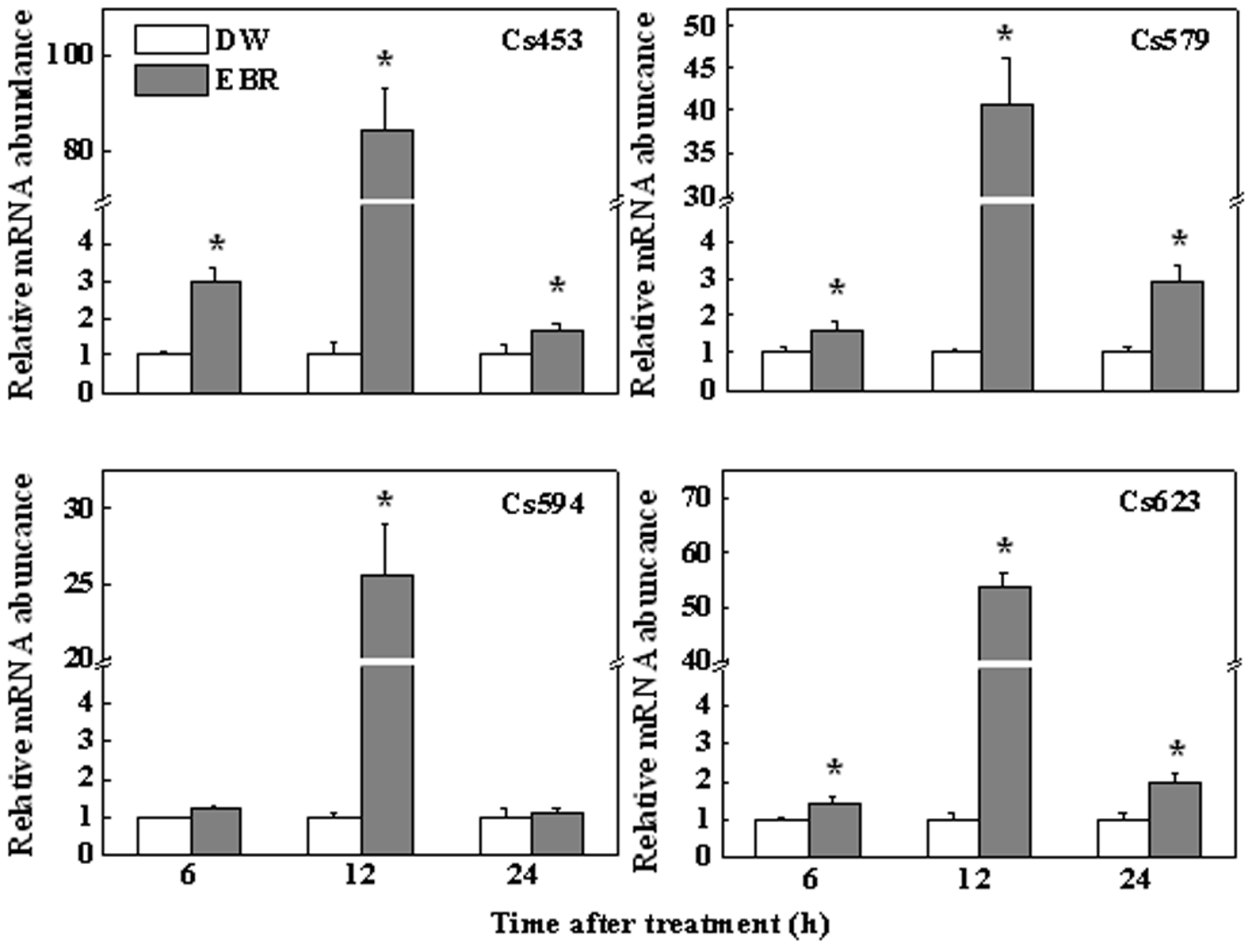
Expression analysis of defense related transcripts obtained from SSH library in cucumber roots. After EBR (0.2 µM) sprayed on mature leaves, roots were harvested at 6, 12, and 24 h after treatment. Relative mRNA abundances of Cs453, Cs579, Cs594 and Cs623 obtained from the SSH library were determined by RT-qPCR analysis. The data were obtained from three separate replicates, and each value in the graph shows means ± SD. Asterisks (*) indicate a significant difference from the untreated control at *P*≤0.05 according to Tukey’s test.

We then examined the EBR-induced changes in the transcripts of genes, Cs453, Cs579, Cs594, and Cs623 identified from the SSH library in the local and systemic leaves (Figure 4) and in the roots (Figure 5). In the local leaves, only a slight increase in the transcript of Cs453 was observed at 3 h after the EBR treatment (Figure 4). However, a 4-fold increase was observed for Cs594 as early as 0.5 h after EBR application, and Cs579 and Cs623 transcripts were upregulated by 7- and 2-fold at 1 h after treatment, respectively. Depending on the genes examined, the transcripts of these genes in the systemic leaves were also altered by the EBR treatment (Figure 4). Transcripts for Cs453 and Cs623 were upregulated by 2-fold in the systemic leaf at 6 and 3 h, respectively. EBR-induced changes in the transcripts for other genes or at other time points were negligible with relative abundance at 0.5∼2-fold. Interestingly, a sharp increase in the abundance of these four genes was observed in the roots. Cs453, Cs579, Cs594 and Cs623 showed a slight elevation at 6 h after the foliar application of EBR; these transcripts were elevated by 80-, 40-, 25-, and 20-fold, respectively, at 12 h, compared with DW treatment, and then declined. All these results suggested that EBR treatment could dynamically increase the transcripts of an array of genes in the phloem.

## Discussion

Over the past several years, genetic and biochemical approaches have yielded significant progress in the understanding of BR signaling. However, the underlying mechanism of the BR regulation of systemic resistance is largely unknown. In this study, we found that EBR induced a systemic tolerance against oxidative stress in cucumber (Figure 1 & 2). This is consistent with our earlier finding that local exposure to EBR led to systemic resistance against oxidative stress in upper and lower leaves and resistance to *Fusarium* pathogen in the roots of cucumber [7]. Several lines of evidence showed that BRs do not undergo long-distance transport between the shoot and roots [6]. By the transcript analysis of BRs biosynthesis genes, we also confirmed the immobility of exogenously applied EBR in plants of cucumber [7]. Accordingly, the systemic stress tolerance in EBR-untreated leaves and roots could be attributed to signal(s) other than EBR. As the phloem is the major route for the communication between source and sink, mRNAs could function as long-distance information macromolecule to control gene expression in remote tissues [24]. Hence, it is of great interest to investigate the changes in the transcriptional profile of the phloem after BRs applied to the source leaves. To our knowledge, this is the first study to address the effects of BRs on the specific mRNA population in the phloem translocation stream.

By constructing an SSH library using the phloem sap mRNA, we identified 14 transcripts out of 260 clones. It should be noted that this up-regulated group represented a very small change in the total population of phloem transcripts, as compared to the transcripts found in the phloem of other cucurbits [16]. Probably, plants have evolved a highly robust mechanism for the exchange of information macromolecules between the companion cell and the sieve tube system [25]. Furthermore, BR-induced transcription has been described as weak and slow in plants [26]. Compared with the induction by other hormones, such as auxin that has been reported to induce transcripts in excess of 10-fold [27], only a very limited numbers of BR-regulated genes have been shown to be induced by more than 2-fold, as based on studies in Arabidopsis [28]–[31]. Hence, it is not surprising that only a small number of transcripts were identified by our SSH library. The RT-qPCR analysis of the expression of these transcripts also supported this notion (Figure 3). As BR-induced transcripts were time-dependent (Figure 2, 4 & 5), we could not exclude the possibility that other important genes were not identified because the samples were taken at different times after EBR treatment in our study. Finally, it is interesting to note that EBR induced much higher transcripts in the distal roots, suggesting a possible amplifying mechanism in the BR signaling cascades in the long distance communication. Probably, it is attributed to the stronger sink for the roots than the systemic leaf in our study because systemic leaves can also carry out photosynthesis.

The mRNAs represented in the subtractive library were related with different cellular functions, including transcriptional regulation, protein translation, transportation and defense responses. Here, we identified four genes that are related with defense response, which imply that they are possibly involved in EBR-regulated systemic resistance. Further analysis of these four transcripts in the local leaves, systemic leaves and roots showed that they were not only induced in the local leaves, but also induced in the systemic leaves and roots (Figure 4, 5). How these genes were involved in the systemic stress tolerance is of great interest. It is worth noting that EBR-induced transcripts were dynamic and transcripts of these genes were even down-regulated at several time points. Although the magnitude of change is negligible in RT-qPCR and microarray analysis, we could not exclude the interference from other BR-induced signaling such as ROS and the involvement of feedback regulation of the gene transcript. There are evidences that foliar EBR application could induce ROS generation in the phloem cells whilst ROS could transiently decrease auxin signaling [7], [32].

In the four genes identified, Cs623 (an MLP-like protein) is known as a phloem defense-related protein, whereas Cs579 possibly functions as a molecular chaperone [33]. Cs594 was identified as a phloem protein 2 that contains an F-box domain, also known as a phloem defense-related protein [34]. Notably, these three genes were first reported as BR-regulated transcripts. Further study should be carried out to confirm their mobility and analyze the functions of these genes. Cs453 encodes IAA14, which is a member of the auxin/indole-3-acetic acid (Aux/IAA) protein family. In a recent study on the transcript profile of melon phloem-sap, Omid and others [23] identified three auxin signaling-related transcripts, two are *Aux/IAA* (F-308 and F-571), and the third one is *small auxin-up RNA* (SAUR, UN-131). An alignment showed that Cs453 is an ortholog of F308. In grafted apple, *MpSLR/IAA14*, was probed to transport a long distance through the graft union [35]. Much research has indicated that there is crosstalk between the BRs and auxins. A microarray analysis of brassinosteroid-regulated genes in Arabidopsis identified several auxin-related genes, which revealed a marked overlap in the BR- and auxin-signaling pathways [28]. Nakamura and others [36] examined the function of Aux/IAA proteins in auxin- and BR-signaling pathways; they found that IAA proteins function as signaling components that modulate BR sensitivity in an organ-dependent manner. The crosstalk between these two hormones probably occurs through the combinatorial regulation of some common target genes, especially the promoters of the target genes [26]. In this regard, IAA14 functions as a transcriptional activator and may interact with a repressor-type ARF to allow the expression of genes repressed by the ARF [37]. Interestingly, BRs are known for their ability in triggering the generation of ROS which are involved in the regulation in IAA14 [5], [7], [38], and both BRs and IAA14 are involved in the regulation of lateral root development [39]. It is, therefore, plausible that Cs453 is one of the candidates for the mediation of a BR-regulated systemic effect through auxin signaling in the long-distance communication.

### Conclusions

By constructing an SSH cDNA library, we identified EBR up-regulated genes in cucumber phloem sap. The further analysis of four cDNAs, which were related with the defense response, suggested that they may be important signaling components functioning in EBR-regulated systemic resistance; in particular, we suggest that Cs453 may be an important candidate. Further study should be carried out to confirm their mobility and analyze the functions of these genes, which will eventually add to our understanding of how BRs regulate the plant systemically.

## Supporting Information

Table S1 Specificity of primers used for RT-qPCR assays.
(DOC)
