# Supplementary material for: Brassinosteroids-Induced Systemic Stress Tolerance was Associated with Increased Transcripts of Several Defence-Related Genes in the Phloem in Cucumis sativus
Source: PLoS One. 2013 Jun 19;8(6):e66582. doi: 10.1371/journal.pone.0066582 (PMC3686678; doi:10.1371/journal.pone.0066582)
Supplement: Table S1 — Specificity of primers used for RT-qPCR assays. (DOC) [file pone.0066582.s001.doc]

**Table S1.** Specificity of primers used for qRT-PCR assays

| **Gene(Clone name)** | **Encoding protein** | **Accession** | **Primer pairs** |
| --- | --- | --- | --- |
|  |  | **Number** |  |
| ***Actin*** | Actin | AAZ74666 | F: TGGACTCTGGTGATGGTGTTA |
|  |  |  | R:CAATGAGGGATGGCTGGAAAA |
| ***CAT*** | Catalase | DQ641067 | F:AAGATGTTGCAAGCGAGAAG |
|  |  |  | R:GGGAGCATTAACAGGCAACT |
| ***cAPX*** | Cytosolic ascorbate peroxidase | EF426538 | F:TCCACCCTGGTAGAGAGGAC |
|  |  |  | R:AATGTCCTGGTCCGAAAGAC |
| ***GR*** | Glutathione reductase | EF530128 | F:CTAAGCGTGTTGTGGTGCTT |
|  |  |  | R:ACTTTGGCACCCATACCATT |
| ***MDAR*** | Monodehydroascorbate reductase | D26392 | F:AGGTTGTGGGAGCTTTCCTA |
|  |  |  | R:AAACCTTGGATGCAAAGGAG |
| **Cs60** | Microtubule binding protein | JG391942 | F:TTTCGCGGGAATCTTAATGT |
|  |  |  | R:TGCAAGACTGGAGTTGAAATG |
| **Cs453** | Auxin-responsive protein IAA14 | JG391949 | F:GCAGGTACCTTGGGAGATGT |
|  |  |  | R:ATCCATCTGTCTTGCCTCCT |
| **Cs418** | tRNA-binding region domain-containing protein | JG391947 | F:GGGATTGATTCGATTCTCTCAT |
|  |  |  | R:TGCTTCCTATCCAAAGCAGA |
| **Cs681** | Yippee putative zinc-binding protein | JG391943 | F:GATGTCCTCATAAAGCGCAA |
|  |  |  | R:TTTCTGGTTACCCATTTCCC |
| **Cs579** | Ankyrin repeat domain-containing protein 2 | JG391948 | F:GGTGCTGAAGTTGCTTGAAA |
|  |  |  | R:AGGCAACTCCGGTCCTAAC |
| **Cs642** | 40S Ribosomal protein S7 | JG391945 | F:GACCCAAAGGAGAGGAACA |
|  |  |  | R:ACGGTTATCGGGAACTCAAAC |
| **Cs594** | F-box protein PP2-A14 | JG391950 | F:GGTGGGTTGAAATAGGAGGA |
|  |  |  | R:TTGTTGTTGGAAACTGAGAGTTC |
| **Cs564** | DNA binding / methylated histone residue binding | JG391944 | F:AAAGCATTTAGGCCCTTGTG |
|  |  |  | R:GCCGAGCACATTAAGCAGT |
| **Cs623** | MLP-like protein 328 | JG391955 | F:GATTGGGACACTCATGGTCA |
|  |  |  | R:CCTTCCAACCCAATCAAAGT |

F indicates forward and R indicates reverse.
